# Influences of the Reaction Temperature and Catalysts on the Pyrolysis Product Distribution of Lignocellulosic Biomass (Aspen Wood and Rice Husk)

**DOI:** 10.3390/polym15143104

**Published:** 2023-07-21

**Authors:** Tanglei Sun, Zhuo Chen, Ruisi Wang, Yantao Yang, Lu Zhang, Yanling Li, Peng Liu, Tingzhou Lei

**Affiliations:** 1 Institute of Urban & Rural Mining, Changzhou University, Changzhou 213164, China; suntanglei@cczu.edu.cn (T.S.); changzhoustudent@163.com (L.Z.);; 2 Changzhou Key Laboratory of Biomass Green, Safe & High Value Utilization Technology, Changzhou 213164, China; 3 School of Management and Economics, North China University of Water Resources and Electric Power, Zhengzhou 450046, China

**Keywords:** reaction temperature, catalyst, aspen wood, rice husk, pyrolysis, product distribution

## Abstract

It is important to clarify the distribution of pyrolysis products from lignocellulosic biomass for its thermal transformation to produce high-quality bio-oil. Influences of the reaction temperature and catalysts on the pyrolysis product distribution from aspen wood (AW) and rice husk (RH) were studied by pyrolysis-gas chromatography/mass spectrometry (Py-GC/MS). The difference in components from the lignocellulosic biomass results in different pyrolysis characteristics of the biomass raw materials. The reaction temperature significantly influences the product distribution from AW and RH pyrolysis. In all AW catalysis experiments, acids (8.35%), ketones (3.79%), phenols (4.73%), and esters (1.50%) have the lowest content while carbohydrates (48.75%) demonstrate the highest content when taking zinc chloride (ZnCl_2_) as the catalyst; the HZSM-5 molecular sieve (HZSM-5) promotes the generation of esters (7.97%) and N-compounds (22.43%) while inhibiting production of aldehydes (2.41%); addition of an MCM-41 molecular sieve (MCM-41) is conducive to increasing the contents of aldehydes (21.29%), furans (5.88%), ketones (22.30%), acids (20.46%), and hydrocarbons (4.85%), while reducing the contents of alcohols (0) and carbohydrates (0). In all RH catalysis experiments, the addition of ZnCl_2_ helps increase the content of carbohydrates (39.16%) and decrease the contents of ketones (3.89%), phenols (5.20%), alcohols (2.34%), esters (1.13%), and N-compounds (3.09%); when applying HZSM-5 as the catalyst, hydrocarbons (18.28%) and alcohols (6.66%) reach their highest content while acids (13.21%) have the lowest content; MCM-41 promotes the generation of aldehydes (25.33%) and furans (5.55%) while inhibiting that of carbohydrates (1.42%).

## 1. Introduction

The excessive use of traditional fossil energy resources has caused global warming, environmental pollution, and an energy crisis [1,2]. How to protect the environment and ensure adequate energy reserves while driving apace development of the global economy has become an active research direction in various countries. China is rich in biomass resources, where the forestry and agricultural residues alone can be used as energy resources, that every year are equivalent to 500 megatons (Mt) of standard coal [3]. The reasonable and efficient utilization of these resources is a bond to tackle energy and environmental problems. Lignocellulosic biomass (equivalent to 370 Mt of standard coal), as the main body of biomass resource in China, mainly includes forestry and agricultural residues and woods, and it is also an object of most concern in the utilization process of biomass energy [4,5,6]. Fast pyrolysis and liquefaction of biomass is a promising biomass utilization technique; it can transform biomass into high-quality bio-oils, which can be partially substituted for fossil-fuel energy [7]. To clarify the product distribution from biomass pyrolysis, many researchers in China and abroad have investigated the three major components of biomass (cellulose, hemicellulose, and lignin) and lignocellulosic biomass [8,9,10,11]. However, there is a wide variety of lignocellulosic biomass, and it yields diverse pyrolysis products. Therefore, it is necessary to examine the product distribution and characteristics of the pyrolysis process from different types of biomasses in forestry and agricultural residues. This is important when trying to ascertain the mechanism of pyrolysis and product utilization of biomass.

The reaction temperature is an extremely important factor influencing the product distribution from biomass pyrolysis [12]. Sun et al. pointed out that the class and yield of pyrolysis products of corn straw at different reaction temperatures show obvious differences and pyrolysis at 600 °C is conducive to increasing the yield of condensable volatile matter during the pyrolysis of corn straw [6]. Qi et al. found that the content of aromatic-rich bio-oils increases monotonically with increasing reaction temperature from 500 to 900 °C by studying the product distribution from the co-pyrolysis of microalgae and polypropylene [13]. Liu et al. considered, as a result of a literature survey, that the pyrolysis of cellulose can be divided into four stages: that occurring below 300 °C, at 300–370 °C, at 370–450 °C, and that occurring above 450 °C; the pyrolysis of hemicellulose can be divided into two stages: below 300 °C and above 300 °C; the pyrolysis of lignin is generally classified into three stages: below 300 °C, 300–360 °C, and above 360 °C [14,15,16]. Therefore, to obtain the basic parameters for the production of high-quality bio-oils from biomass pyrolysis, it is necessary to select the reaction temperatures based on pyrolysis intervals of the three components to conduct rapid pyrolysis experiments on the typical lignocellulosic biomass.

At present, the bio-oils produced by pyrolysis face problems, including high water and oxygen contents, low heat value, and poor stability [17,18,19]. The quality of bio-oils needs to be further improved. Catalytic fast pyrolysis, which can improve the quality of bio-oils at the source of pyrolysis, reduces the complexity and difficulty in subsequent quality-improvement and modification processes. Experiments have proven that pyrolysis product distribution can be orientationally controlled by adjusting the content of inorganic salts in the biomass or adding catalysts to the pyrolysis process, thus increasing the yield of the target products while inhibiting the generation of non-target products [20,21,22,23]. Lu et al. found that the contents of products, including levoglucosan (LG), glycolaldehyde (GA), and hydroxyacetone (HA) from cellulose pyrolysis, decline after adding Zn^2+^, while proportions of substances, such as furfural (FF), 5-methylfurfural (ML), formic acid (FA), and acetic acid (AA) increase significantly. In addition, a high Zn^2+^ content can promote the removal and transformation of oxygen compounds and the dehydration of cellulose, thereby improving the quality of bio-oils [24,25]. Xu et al. stated that adding a HZSM-5 molecular sieve (HZSM-5) can control the content of aromatic-rich bio-oils in the products from eucalyptus pyrolysis, and the yield of aromatic hydrocarbons increases significantly with the catalyst ratio [26]. Through lignin pyrolysis at 600 °C using an MCM-41 molecular sieve (MCM-41), Jackson et al. found that the coke yield declines from 41% without adding the catalyst to about 36%. Oxygen-containing aromatic compounds, including phenols and benzodioxofuran are mostly transformed into naphthalene. Meanwhile, the catalyst also promotes the generation of H_2_, CH_4_, and CO, which greatly increases the yields and heat values of bio-oils and biomass gases [27]. Therefore, it is necessary to study the influences of the above three catalysts on the distribution of pyrolysis products from typical lignocellulosic biomass. 

In the present work, the reaction temperatures were selected based on the pyrolysis intervals of biomass components and zinc chloride (ZnCl_2_), HZSM-5, and MCM-41 were used as the catalysts. In conjunction with pyrolysis-gas chromatography/mass spectrometry (Py-GC/MS), influences of the temperature and catalyst on pyrolysis products from typical lignocellulosic biomass (aspen wood (AW) and rice husk (RH)) were analyzed on-line. This provides a reference for the production of bio-oils with a high added value through oriented biomass pyrolysis.

## 2. Experimental Materials and Methods

### 2.1. Experimental Raw Materials

AW and RH were collected from the outskirts of Zhengzhou City, Henan Province, China. They were crushed to a particle size below 80 mesh using a straw crusher, and then dried at 105 °C for 24 h for later use. ZnCl_2_ (AR, 98%) used in the experiments was purchased from Shanghai Macklin Biochemical Co., Ltd. (Shanghai, China), while HZSM-5 (Si/Al = 46, Brunauer–Emmett–Teller [BET] surface area: 350 m^2^/g) and MCM-41 (Si/Al = 25, BET surface area: 750 m^2^/g) were provided by Nankai University Catalyst Co., Ltd. (Tianjin, China) The proximate analysis was performed in accordance with GB/T 28731-2012 [28]. The C and H contents in the ultimate analysis were measured according to GB/T28734-2012 [29], and the N and S contents were analyzed based on GB/T30728-2014 [30] and GB/T28732-2012 [31], individually. Moreover, the O content was calculated by differences. The contents of cellulose, hemicellulose, and lignin in AW and RH were analyzed using the Van Soest method. The experiments were conducted in triplicate to ensure the accuracy of the results.

### 2.2. Sample Preparation

In general, catalysts, such as molecular sieves, can be mechanically mixed with biomass, and inorganic salts are usually added to the raw materials by soaking absorption. AW and RH were separately blended with catalysts (HZSM-5 and MCM-41) at the mass ratios of 3:1, 1:1, and 1:3. After uniform grinding, the mixture was pressed into sheets and then ground. The step was repeated three times to ensure uniform mixing of samples. The samples were cited below according to the raw material, catalyst, and mixing ratio of the catalyst. For example, “AWH31” represents that AW and HZSM-5 were mixed at the mass ratio of 3:1. ZnCl_2_ of 30, 75, 150, and 300 mg was added to 30 mL of deionized water, followed by the addition of 3 g of AW and RH to each solution. After stirring the aforementioned solutions at room temperature for 2 h, they were oven-dried at 105 °C to constant mass. The abbreviations of samples are summarized thus: AWZn30 means that AW was soaked in deionized water in which 30 mg of ZnCl_2_ was added (other abbreviations in Table 1 have similar meanings). The contents of metal ions in the raw materials were measured following the general rules for inductively coupled plasma-atomic emission spectrometry (JY/T015-1996) [6].

### 2.3. Rapid Pyrolysis by Py-GC/MS

Based on the pyrolysis temperature intervals of the biomass components, AW and RH were separately pyrolyzed at six temperatures including 285, 345, 445, 500, 600, and 700 °C for 10 s. Catalytic rapid pyrolysis experiments were all conducted at 500 °C for 10 s. The experimental apparatus was a double-screw pyrolyzer (EGA/PY-3030D, Frontier Lab, Koriyama, Japan). About 0.1 mg of the sample was put into a sample cup, in which quartz wool was placed on two sides to prevent solid particles from spilling. The gas produced after pyrolysis entered the GC/MS (QP2010 Ultra, Shimadzu, Kyoto, Japan) for real-time online analysis and the temperature of the injection port was 250 °C. Chromatography was carried out using an Rtx-5MS capillary column (Restek; length: 30 m; internal diameter: 250 μm; membrane thickness: 0.25 μm). Taking high-purity helium (He, 99.999%) as the carrier gas, the column flow and split ratio were, respectively, 1.27 mL/min and 100:1. The gas phase heating program was set as follows: the initial furnace temperature of 50 °C, heat preservation for 5 min, temperature rise to 260 °C at 10 °C/min, and heat preservation for 10 min. The interface temperature of GC/MS was 280 °C and the ion source was kept at 230 °C. The spectral range was set to be 35 ≤ *m*/*z* ≤ 500. Various chromatographic peaks were determined according to spectra in NIST11 and F-Search PY-1110E-181 spectral libraries and previous research data [4,6].

The chromatographic peak area of each compound in the Py-GC/MS pyrolysis products is directly proportional to its concentration. Hence, changes in the yield of various compounds can be reflected by comparing the average peak areas under different reaction conditions. The area percentage can be used to elucidate changes in the relative content [32,33].

## 3. Results and Discussion

### 3.1. Chemical Analysis of Lignocellulosic Biomass

Results from the proximate analysis, elemental analysis, and component analysis of the AW and RH in Table 2 show that the two types of biomasses differ slightly in the contents of water and fixed carbon while exhibiting significant differences in the ash and volatile contents. AW contains a higher volatile content with a lower ash content, while RH has a higher ash content with a lower volatile content. The two types of biomasses both mainly contain C, H, and O, while containing low contents of N and S. Component analysis implies that cellulose accounts for the largest proportion in AW, followed by lignin and hemicellulose. The proportions of cellulose and hemicellulose are high, while the proportion of lignin is low in RH. The discrepancies in the biomass components will cause different types of biomass raw materials to show distinct pyrolysis characteristics, which is finally shown as the large difference in the pyrolysis product distribution.

### 3.2. Influence of the Reaction Temperature on the Product Distribution from AW and RH Pyrolysis

**Figure 1:**
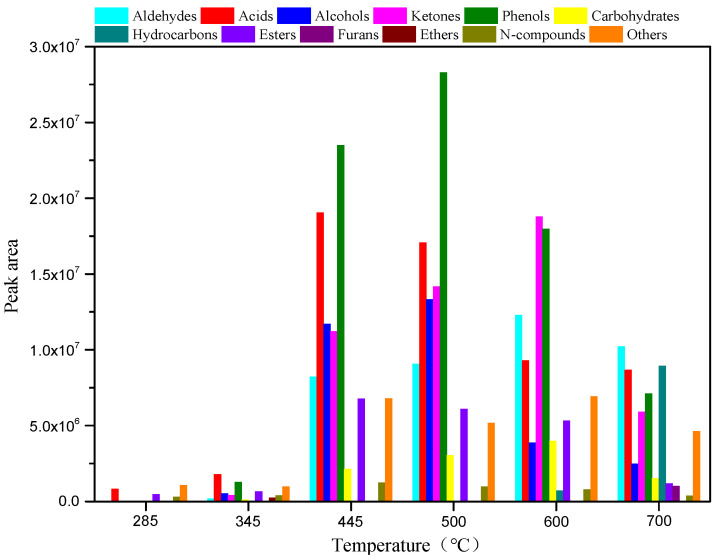
Yields of various compounds in AW pyrolysis bio-oil at temperature ranging between 285 and 700 °C.

**Figure 2:**
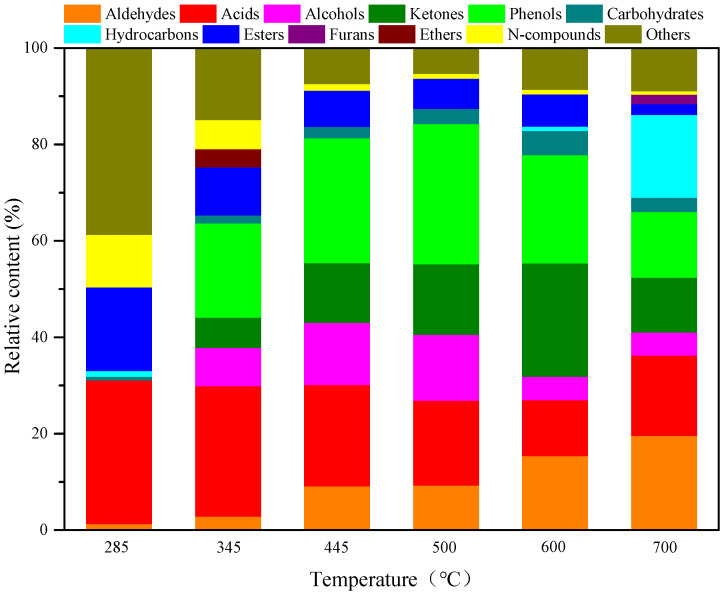
Product distributions of AW pyrolysis bio-oils at different temperatures.

To reveal the relationship between the structure and pyrolysis products of AW, AW was pyrolyzed for 10 s at different reaction temperatures with no catalysts. The pyrolysis product distribution is illustrated in Figure 1 and Figure 2. As shown in Figure 1, AW pyrolysis produces diverse types of compounds, which can be classified into aldehydes, acids, alcohols, ketones, phenols, carbohydrates, hydrocarbons, esters, furans, ethers, and N-compounds. In addition, the total ion chromatographs of AW pyrolysis products contained hundreds of peaks, some of which were not identifiable. These were classified as “others”. The total peak areas at each temperature in the range of 285–700 °C were determined to be 2.77 × 10^6^, 6.61 × 10^6^, 9.07 × 10^7^, 9.73 × 10^7^, 8.00 × 10^7^, and 5.21 × 10^7^ by adding the peak areas of various compounds. Figure 1 demonstrates that a pyrolysis temperature of 500 °C is conducive to increasing the content of bio-oils and the yields of all compounds vary significantly with changes in the pyrolysis temperature. As the temperature is increased from 285 to 600 °C, the yields of aldehydes and carbohydrates show gradual increases and then begin to decline as the temperature is increased from 600 to 700 °C. In the experimental temperature range, the yields of acids, esters, and N-compounds increase at first, and then decrease as the pyrolysis temperature rises, and they all reach their maxima at 445 °C. Alcohols and phenols are not produced at a low temperature of 285 °C, while their yields tend to increase at first, and then decline as the temperature rises in the range of 345–700 °C, reaching their maximum at 500 °C. Similar to alcohols and phenols, no ketones are detected at 285 °C, while their yield increases at first, and then decreases when the temperature rises from 345 to 700 °C, and it reaches the maximum at 600 °C. Hydrocarbons are only produced at 285 °C, 600 °C, and 700 °C. At the low temperature of 285 °C, what is produced is a low content of alkanes; traces of olefins are generated at 600 °C. The yield of hydrocarbons is the highest at 700 °C; these mainly include olefins and aromatic hydrocarbons. Small amounts of ethers are produced only at 345 °C, while furans are only generated at 700 °C. As displayed in Figure 2, many other substances are produced at the pyrolysis temperature of 285 °C, the content of which reaches 38.74%. Apart from this, acids are the most important pyrolysis products at 285 °C, with a relative content of 29.74%; esters and N-compounds also account for large proportions (17.31% and 10.87%). As the temperature rises to 345 °C, acids and phenols become the main pyrolysis products at the temperature and their contents, respectively, reach 27.09% and 19.52%. With a further temperature rise to 445–500 °C, phenols become the predominant products and their relative contents reach 25.92% and 29.08%, while the content of acids remains relatively high, being 21.03% and 17.59%. At a pyrolysis temperature of 600 °C, the proportions of ketones and aldehydes are enlarged while that of acids are reduced, so that ketones, phenols, and aldehydes become the most important pyrolysis products, with contents, respectively, of 23.50%, 22.48%, and 15.37%. At 700 °C, the proportion of phenols declines and the deoxygenation of oxygen compounds at high temperatures promotes the production of a greater number of hydrocarbons, the proportion of which reaches 17.17%. Aldehydes predominate (19.59%) at that temperature, while acids are also present in significant amounts (16.66%).

**Figure 3:**
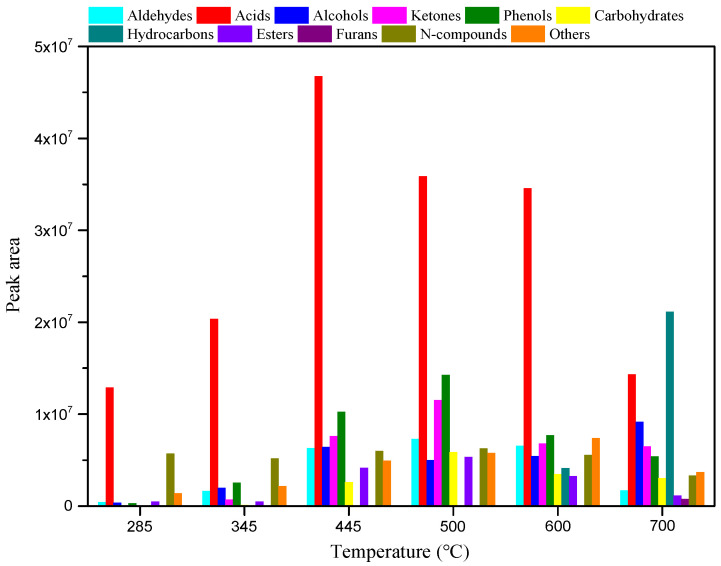
Yields of various compounds in RH pyrolysis bio-oil between 285 and 700 °C.

**Figure 4:**
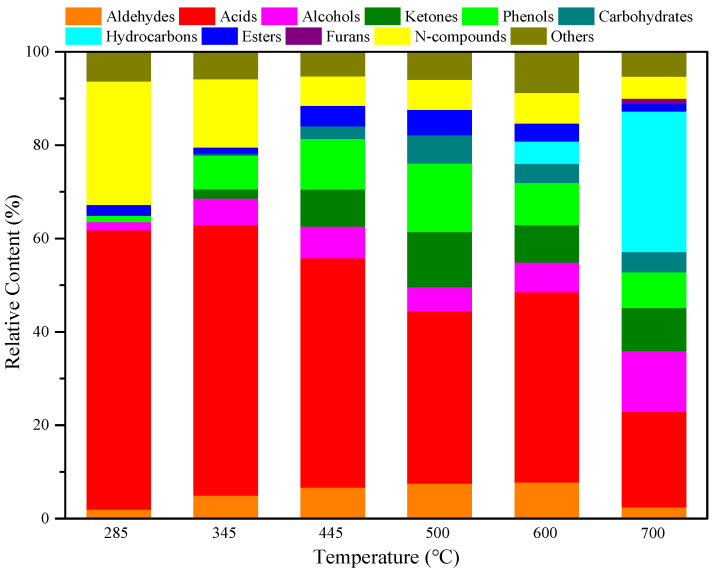
Product distributions of RH pyrolysis bio-oils at different temperatures.

To clarify the relationship between the structure and pyrolysis products of RH, RH was pyrolyzed for 10 s at different temperatures without adding catalysts. The pyrolysis product distribution is illustrated in Figure 3 and Figure 4. As shown in Figure 3, diverse compounds produced from RH pyrolysis can be divided into aldehydes, acids, alcohols, ketones, phenols, carbohydrates, hydrocarbons, esters, furans, and N-compounds. After the summation of peak areas of various compounds in Figure 3, the total peak areas at each temperature in the range of 285–700 °C are obtained as 2.15 × 10^7^, 3.51 × 10^7^, 9.50 × 10^7^, 9.71 × 10^7^, 8.48 × 10^7^, and 7.01 × 10^7^. Figure 3 also shows that the pyrolysis temperature in the range of 445–500 °C is conducive to improving the yield of bio-oils, and the yields of all compounds vary significantly as the pyrolysis temperature changes. As the temperature is increased from 285 to 500 °C, the yields of aldehydes, phenols, and esters increase, while they begin to decline as the temperature is increased from 500 to 700 °C. The yield of acids increases at first and then reduces with the rise of the pyrolysis temperature in the experimental range, and it reaches the maximum at 445 °C. The maximum yields of alcohols and N-compounds are obtained separately at 700 and 500 °C. Ketones and carbohydrates are not detected at 285 °C and their yields increase, and then decrease as the temperature rises from 345 to 700 °C, reaching the maximum at 500 °C. Hydrocarbons are only produced at 600–700 °C and their yield increases with increasing temperature, and they always include olefins, alkyne, and aromatic hydrocarbons, whereas, furans are only generated at a high temperature of 700 °C. As illustrated in Figure 4, acids are the main pyrolysis products at 285 °C, with a relative content of 59.79%, and N-compounds also account for a large proportion at 26.42%. As the temperature increases to 345 °C, acids and N-compounds are still the most important pyrolysis products and their contents are, respectively, 58.00% and 14.67%. With the rise of pyrolysis temperature to 445 °C, the content of N-compounds reduces while that of phenols increases; acids and phenols are major pyrolysis products under this condition, separately accounting for 49.16% and 10.80%. At the pyrolysis temperature of 500 °C, the content of acids declines to some extent; acids (36.95%), phenols (14.67%), and ketones (11.84%) represent three predominant compounds. Akin to the case at 285–500 °C, acids are also the compounds of the highest content at 600 °C and their content can reach 40.79%. With a further rise of the pyrolysis temperature to 700 °C, deoxygenation of oxygen compounds at high temperatures facilitates the generation of a large number of hydrocarbons, so hydrocarbons are compounds of the largest content (30.12%) under this condition; acids and alcohols also have high contents of 20.41% and 13.04%, respectively.

The above results show that the types and yields of compounds generated by the non-catalytic pyrolysis of AW and RH were influenced significantly by temperature. In addition, the compositional and structural differences between these two types of biomasses led to marked differences in product distributions in the pyrolysis process. As provided in the Supplementary Data (Tables S1 and S2), the types of pyrolytic compounds of AW and RH increased and the molecular structures of those tended to be simpler with increasing temperature from 285 °C to 700 °C. After a sequence of reactions including depolymerization, cracking, dehydration, deoxygenation, decarbonylation, and decarboxylation under high temperatures, the macromolecular oxygenates and N-compounds were converted into small molecular compounds. Moreover, higher temperatures can remove certain oxygen-containing functional groups to obtain hydrocarbons or promote the dehydration, oligomerization, and aromatization of small molecules to generate aromatics [4].

### 3.3. Effects of Various Catalysts and Their Concentration on Product Distribution from AW Pyrolysis

**Figure 5:**
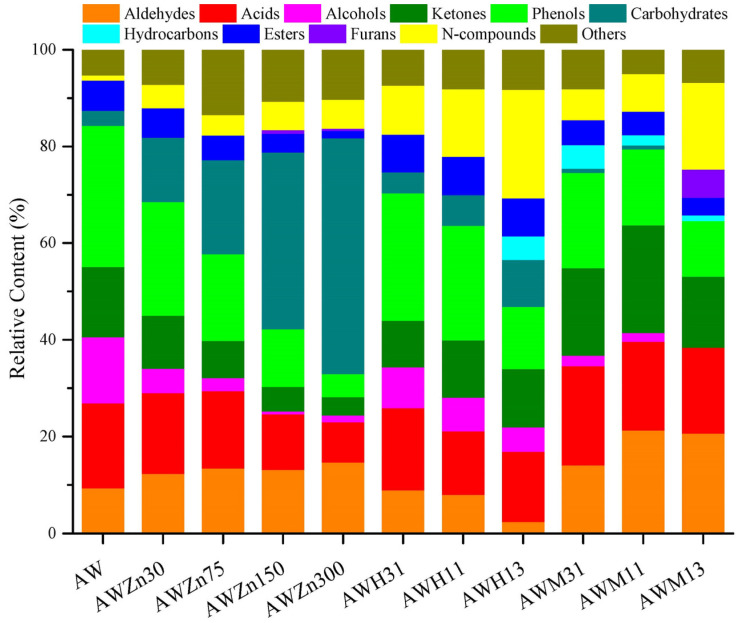
Product distributions of AW catalytic fast pyrolysis.

**Figure 6:**
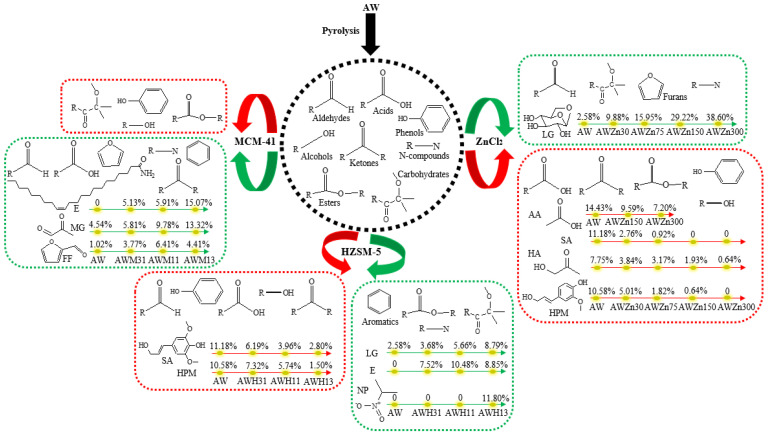
Influences of three catalysts on the product distribution of AW pyrolysis (green represents a promoting effect, red represents an inhibitory effect).

Three different catalysts (i.e., ZnCl_2_, HZSM-5, and MCM-41) were used to perform catalytic rapid pyrolysis experiments on AW, and the results are shown in Figure 5 and Figure 6. Data in Figure 5 indicate that adding ZnCl_2_ promotes the generation of aldehydes, the content of which increases with increasing amounts of catalyst. When using ZnCl_2_ as the catalyst, the contents of most oxygen compounds decrease, including acids, alcohols, ketones, phenols, and esters. The addition of a high content of ZnCl_2_ significantly inhibits the production of acids; within the experimental range, the acid content can be reduced by up to 9.24%. Variation in the content of acids is mainly shown as the reduction of the AA content, which was reduced by 7.23% (Figure 6). This is consistent with previous studies, wherein it was found that ZnCl_2_ supplementation can reduce the AA content of corn stalk pyrolysis products by up to 6.19% [6]. These show that ZnCl_2_ can inhibit the formation of AA in biomass pyrolysis products. In addition, ZnCl_2_ can significantly reduce the alcohol content, up to 13.08% (Figure 5). Changes in the content of alcohols are mainly reflected by the decreased content of *trans*-sinapyl alcohol (SA), the content of which can be reduced from 11.18% to 0 (Figure 6). As displayed in Figure 5, when adding ZnCl_2_, the content of ketones and phenols also reduces by up to 10.78% and 24.35%, respectively (Figure 5). In detail, the reduction of the content of ketones is manifested in a decreased content of HA from 7.75% (AW) to 0.64% (AWZn300) (Figure 6). The decrease in the content of phenols is mainly reflected by the reduced content of 4-((1*E*)-3-hydroxy-1-propenyl)-2-methoxyphenyl (HPM) from 10.58% (AW) to 0 (AWZn300) (Figure 6). The contents of alcohols, ketones, and phenols also decrease significantly when the added amount of ZnCl_2_ is high, which indicates that ZnCl_2_ may change the main path of pyrolysis-product generation for AW. As shown in Figure 5, ZnCl_2_ can significantly increase the content of carbohydrates from 3.14% without adding the catalyst to 48.75% (AWZn300). Variation of the content of carbohydrates is mainly mirrored by the increased content of LG from 2.58% (AW) to 38.60% (AWZn300) (Figure 6). This shows that ZnCl_2_ can make the pyrolysis reaction of AW produce more carbohydrates (especially LG), which is consistent with previous studies; ZnCl_2_ supplementation can improve the LG content of corn stalk pyrolysis products by up to 18.16% [6]. ZnCl_2_ does not promote the generation of hydrocarbons from AW pyrolysis, so the yield of hydrocarbons is always zero. If adding high contents of ZnCl_2_ (AWZn150 and AWZn300), it promotes the production of trace amounts of furans. Moreover, ZnCl_2_ can also suppress the generation of esters and facilitate the production of N-compounds. Such inhibiting or promoting effects become increasingly obvious with increasing amounts of catalyst. In all catalysis experiments, the lowest contents of acids (8.35%), ketones (3.79%), phenols (4.73%), and esters (1.50%) and the highest content of carbohydrates (48.75%) are always determined in the case of AWZn300.

As shown in Figure 5, taking HZSM-5 as the catalyst can apparently decrease the contents of aldehydes, acids, and alcohols, which reduce more remarkably with increasing amounts of catalyst added. Therein, changes in the content of alcohols are manifested as the content of SA declines from 11.18% (AW) to 6.19% (AWH31), 3.96% (AWH11), and 2.80% (AWH13) (Figure 6). The content of ketones is also inhibited by HZSM-5, which is more obvious when the added amount of catalyst is low. HZSM-5 can significantly reduce the content of phenols from 29.08% without adding the catalyst to 26.33% (AWH31), 23.67% (AWH11), and 12.88% (AWH13) (Figure 5). Variation of the content of phenols is mainly reflected by the reduction of the HPM content from 10.58% (AW) to 7.32% (AWH31), 5.74% (AWH11), and 1.50% (AWH13) (Figure 6). High amounts of HZSM-5 (AWH13) may promote the generation of aromatic hydrocarbons, increasing their content from 0 to 4.81% (Figure 5). The production of aromatic hydrocarbons is probably because HZSM-5 can facilitate deoxygenation and promote the transformation of phenolic substances to aromatic hydrocarbons in the pyrolysis. HZSM-5 can promote the generation of carbohydrates, and such an effect becomes increasingly significant with increasing amounts of catalyst. The increased content of carbohydrates is manifested in the increase in the LG content from 2.58% (AW) to 3.68% (AWH31), 5.66% (AWH11), and 8.79% (AWH13) (Figure 5 and Figure 6). It can be seen from Figure 5 that HZSM-5 can slightly increase the content of esters, which is not affected to any significant extent by the amount of catalyst added. HZSM-5 does not promote the generation of furans during AW pyrolysis. Additionally, taking HZSM-5 as the catalyst can significantly augment the content of N-compounds from 1.01%, without adding the catalyst, to 10.14% (AWH31), 13.93% (AWH11), and 22.43% (AWH13). The increased content of N-compounds is mainly reflected by the increased contents of erucamide (E) and 2-nitropropane (NP). The contents of E and NP separately grow from 0 (AW) to 7.52% (AWH31), 10.48% (AWH11), and 8.85% (AWH13), and from 0 (AW) to 11.80% (AWH13) (Figure 6). In all catalysis experiments, the lowest content of aldehydes (2.41%) and the highest content of N-compounds (22.43%) were both obtained in the case of AWH13, while the highest content of esters (7.97%) was produced under AWH11.

As shown in Figure 5, the content of aldehydes rises significantly from 9.32%, without adding the catalyst, to 14.13% (AWM31), 21.29% (AWM11), and 20.64% (AWM13) when adding MCM-41 as the catalyst. Changes in the content of aldehydes are manifested as variations in methylglyoxal (MG) and FF contents: the content of MG grows from 4.54% (AW) to 5.81% (AWM31), 9.78% (AWM11), and 13.32% (AWM13); that of FF increases from 1.02% (AW) to 3.77% (AWM31), 6.41% (AWM11), and 4.41% (AWM13) (Figure 6). MCM-41 can promote the generation of acids, while the promoting effect becomes increasingly attenuated with increasing amounts of catalyst. Addition of MCM-41 results in a significant reduction of the content of alcohols from 13.68%, without adding the catalyst, to 2.15% (AWM31), 1.78% (AWM11), and 0 (AWM13). Changes in the content of alcohols are manifested in the reduction of the SA content from 11.18% (AW) to 0 (AWM31, AWM11, and AWM13). Figure 5 also shows that adding low amounts of MCM-41 (AWM31 and AWM11) can promote the production of ketones. In addition, MCM-41 reduces the content of phenols remarkably from 29.08% (AW) to 19.66% (AWM31), 15.68% (AWM11), and 11.50% (AWM13). Variation of the content of phenols is mainly reflected by the reduction of the HPM content from 10.58% (AW) to 0 (AWM31, AWM11, and AWM13). As illustrated in Figure 5, MCM-41 decreases the generation of carbohydrates and esters, which becomes increasingly apparent as the added amount of the catalyst increases. The presence of MCM-41 promotes the generation of hydrocarbons from AW pyrolysis, while the content of hydrocarbons tends to decrease with increasing addition of the catalyst. Furans are only produced under conditions of a high amount of catalyst (AWM13) and their content can reach 5.88%. Taking MCM-41 as the catalyst can significantly increase the content of N-compounds. In particular, when adding a large amount of the catalyst, the content of N-compounds grows from 1.01% (AW) to 17.89% (AWM13). Changes in the content of N-compounds are mainly reflected by the increasing content of E from 0 (AW) to 5.13% (AWM31), 5.91% (AWM11), and 15.07% (AWM13) (Figure 6). In all catalysis experiments, AWM13 leads to the highest contents of aldehydes (20.64%) and furans (5.88%), with the lowest contents of alcohols (0) and carbohydrates (0). The highest content of ketones (22.30%) is obtained under AWM11, while those of acids (20.46%) and hydrocarbons (4.85%) are attained in the case of AWM31.

### 3.4. Effects of Various Catalysts and Their Concentration on Product Distribution from RH Pyrolysis

**Figure 7:**
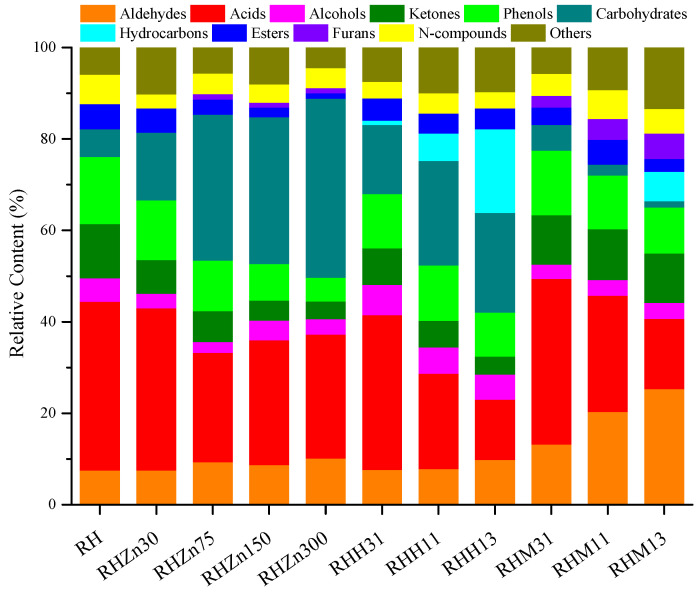
Product distributions of RH catalytic rapid pyrolysis.

**Figure 8:**
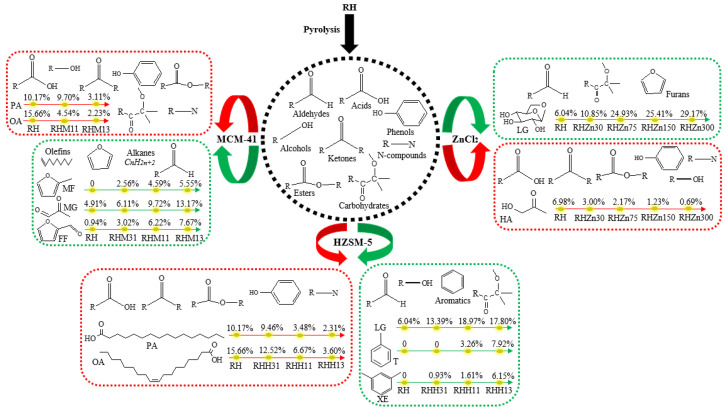
Influences of three catalysts on the product distribution of RH pyrolysis (green represents a promoting effect, red represents an inhibitory effect).

Catalytic rapid pyrolysis experiments of RH were conducted using three different catalysts including ZnCl_2_, HZSM-5, and MCM-41, and the results are illustrated in Figure 7 and Figure 8. Data in Figure 7 indicate that the addition of ZnCl_2_ can promote the generation of aldehydes while inhibiting the production of alcohols and N-compounds, whereas, the contents of the three compounds do not change in any particular way with the increased amount of added catalyst. Adding ZnCl_2_ can apparently reduce the generation of acids from 36.95% without adding the catalyst to 35.50% (RHZn30), 23.93% (RHZn75), 27.27% (RHZn150), and 27.10% (RHZn300). Changes in the content of acids are mainly shown in the reduction of the content of multiple acids. In addition, taking ZnCl_2_ as the catalyst can also decrease the contents of ketones, phenols, and esters, and their contents reduce more apparently as the amount of catalyst added increases. Variation of the content of ketones is mainly reflected by changes in the HA content, which decreases from 6.98% (RH) to 3.00% (RHZn30), 2.17% (RHZn75), 1.23% (RHZn150), and 0.69% (RHZn300) (Figure 8). Variation of the content of phenols is manifested in the reduction of the contents of multiple phenols. The presence of ZnCl_2_ can promote the generation of carbohydrates from 6.04%, without adding the catalyst, to 14.80% (RHZn30), 31.88% (RHZn75), 32.09% (RHZn150), and 39.16% (RHZn300) (Figure 7). Variation in the content of carbohydrates is mainly reflected by the increment of the LG content from 6.04% (RH) to 10.85% (RHZn30), 24.93% (RHZn75), 25.41% (RHZn150), and 29.17% (RHZn300) (Figure 8). As shown in Figure 7, ZnCl_2_ does not promote the generation of hydrocarbons during RH pyrolysis, so the content of hydrocarbons always remains 0. The addition of high amounts of ZnCl_2_ (RHZn75, RHZn150, and RHZn300) facilitates the generation of slight furans and variation in the added amount of catalyst does not influence the furan content to any significant extent. In all catalysis experiments, the lowest contents of ketones (3.89%), phenols (5.20%), and esters (1.13%) and the highest content of carbohydrates (39.16%) are obtained under RHZn300. Separately, RHZn75 and RHZn30 result in the lowest contents of alcohols (2.34%) and N-compounds (3.09%).

As shown in Figure 7, adding a high amount of HZSM-5 (RHH13) can promote the production of aldehydes, while the content of acids declines significantly in the presence of HZSM-5 from 36.95%, without adding the catalyst, to 33.82% (RHH31), 20.89% (RHH11), and 13.21% (RHH13). Variation in the content of acids is manifested in the reduction in contents of palmitic acid (PA) and oleic acid (OA). The former reduces from 10.17% (RH) to 9.46% (RHH31), 3.48% (RHH11), and 2.31% (RHH13), while the latter declines from 15.66% (RH) to 12.52% (RHH31), 6.67% (RHH11), and 3.60% (RHH13) (Figure 8). Data in Figure 7 also show that adding a low amount of HZSM-5 (RHH31) can augment the content of alcohols (albeit slightly), whereas, HZSM-5 suppresses the generation of ketones and phenols, and the inhibitory effect becomes increasingly prominent with the increasing amounts of catalyst. Using HZSM-5 as the catalyst can promote the generation of carbohydrates from 6.04% (RH) to 15.09% (RHH31), 22.85% (RHH11), and 21.84% (RHH13). Changes in the content of carbohydrates are mainly reflected by the variation of the LG content, which grows from 6.04% (RH) to 13.39% (RHH31), 18.97% (RHH11), and 17.80% (RHH13) (Figure 8). As displayed in Figure 7, the addition of HZSM-5 significantly enlarges the content of hydrocarbons (aromatic hydrocarbons) from 0 (RH) to 0.93% (RHH31), 5.96% (RHH11), and 18.28% (RHH13). Variation in the content of hydrocarbons is mainly shown in the increased contents of toluene (T) and m-xylene (XE). The former grows from 0 (RH) to 3.26% (RHH11) and 7.92% (RHH13), while the latter rises from 0 (RH) to 0.93% (RHH31), 1.61% (RHH11), and 6.15% (RHH13) (Figure 8). HZSM-5 also plays a role in reducing the production of esters, while the effect is not significant. Moreover, HZSM-5 does not promote the generation of furans during RH pyrolysis, so the furan content is always zero. In addition, the presence of HZSM-5 decreases the content of N-compounds, while changes in the amount of added catalyst do not apparently affect the content thereof (Figure 7). In all catalysis experiments, alcohols reach their highest concentration (6.66%) under RHH31, while RHH13 leads to the lowest content of acids (13.21%) and the highest content of hydrocarbons (18.28%).

As illustrated in Figure 7, the content of aldehydes can be increased from 7.50% (RH) to 13.18% (RHM31), 20.33% (RHM11), and 25.33% (RHM13) when using MCM-41 as the catalyst. Changes in the content of aldehydes are mainly shown as increments of the MG and FF contents. The former grows from 4.91% (RH) to 6.11% (RHM31), 9.72% (RHM11), and 13.17% (RHM13), and the latter increases from 0.94% (RH) to 3.02% (RHM31), 6.22% (RHM11), and 7.67% (RHM13) (Figure 8). MCM-41 with a high adding amount can significantly reduce the content of acids from 36.95% without adding the catalyst to 25.40% (RHM11) and 15.38% (RHM13) (Figure 7). Variation in the content of acids is manifested in the reduction of the PA and OA contents. The former decreases from 10.17% (RH) to 9.70% (RHM11) and 3.11% (RHM13), and the latter decreases from 15.66% (RH) to 4.54% (RHM11) and 2.23% (RHM13) (Figure 8). As displayed in Figure 7, MCM-41 can inhibit the generation of alcohols while changes in the amount added do not significantly affect the content of alcohols. The presence of MCM-41 slightly decreases the content of ketones; at the same time, it suppresses the production of phenols and carbohydrates, and the contents of the two reduce more significantly with the increasing addition of the catalyst. Hydrocarbons are only produced when adding a larger amount of the catalyst (RHM13) and their content rises from 0 (RH) to 6.38% (RHM13); moreover, MCM-41 also reduces the generation of esters and N-compounds, the contents of which, however, are unaffected by the amount of the catalyst added. The presence of MCM-41 promotes the generation of 2-methylfuran (MF), so that the MF content increases from 0 (RH) to 2.56% (RHM31), 4.59% (RHM11), and 5.55% (RHM13) (Figure 8). In all catalysis experiments, the highest contents of aldehydes (25.33%) and furans (5.55%), while the lowest content of carbohydrates (1.42%) are all attained in the case of RHM13.

According to the aforementioned studies on catalytic pyrolysis of two types of biomasses, the compositional and structural differences between AW and RH led to marked differences in product distributions in the pyrolysis process. In addition, the difference in catalyst performance also had different effects on the distribution of the pyrolysis products of biomass. The above results show that ZnCl_2_ has no effect on the formation of hydrocarbons during AW and RH pyrolysis but can significantly change the distribution of oxygen-containing compounds. ZnCl_2_ can increase the contents of aldehydes, carbohydrates, and furans while reducing the contents of acids, alcohols, ketones, phenols, and esters. Changes in the relative amount of carbohydrates are mainly manifested in the change in the proportion of LG. These catalytic effects are more obvious at higher doses. This is consistent with previous research [6].

The addition of HZSM-5 is conducive to the formation of carbohydrates and aromatic hydrocarbons during the pyrolysis of AW and RH, while suppressing the production of oxygen-containing compounds such as acids, ketones, and phenols. This may be because the acidity of HZSM-5 promoted deoxygenation and facilitated the conversion of phenols to aromatic hydrocarbons during pyrolysis. The formation of aromatic hydrocarbons may be related to the polymerization of alkenes or alkynes, or to the condensation of compounds containing C=O [26].

MCM-41 can improve the selectivity of aldehydes, furans, and hydrocarbons, while also decreasing the generation of alcohols, phenols, carbohydrates, and esters during the pyrolysis of AW and RH. Changes in the relative amounts of aldehydes, furans, and carbohydrates were mainly manifested in changes in the proportions of MG, FF, MF, and LG, respectively. MG, FF, and MF are valuable products that can be converted into other useful chemicals. They are typically derived from hemicellulose or produced by the dehydration of carbohydrates, mainly LG, which are derived from cellulose and hemicellulose pyrolysis [4]. Compared with HZSM-5, MCM-41 had a larger specific surface area, pore volume, and average pore diameter, which were conducive to the provision of sufficient reaction sites and selectivity. Therefore, MCM-41 can also promote several deoxygenation reactions that convert oxygenates into olefins and aromatics.

## 4. Conclusions

This study on the distribution of pyrolysis products of AW and RH under different reaction temperatures and catalysts provides important references for clarifying the distribution of biomass pyrolysis products and preparing high-quality bio-oil directionally. The reaction temperature and catalysts significantly influence the product distribution from AW and RH pyrolysis: the difference in the components of lignocellulosic biomass results in different pyrolysis characteristics of biomass raw materials. The pyrolysis temperature of 500 °C is conducive to the generation of condensable volatile matter from AW pyrolysis. Acids and phenols are the main products arising from AW pyrolysis. In all AW catalysis experiments, acids, ketones, phenols, and esters have the lowest contents while carbohydrates are present in the greatest amounts when taking ZnCl_2_ as the catalyst; HZSM-5 promotes the generation of esters and N-compounds while inhibiting the production of aldehydes; addition of MCM-41 is conducive to increasing the contents of aldehydes, furans, ketones, acids, and hydrocarbons while reducing the contents of alcohols and carbohydrates.

Pyrolysis temperatures in the range of 445–500 °C are conducive to increasing the yield of condensable volatile matter from RH pyrolysis. Acids are the primary product from RH pyrolysis. In all RH catalysis experiments, addition of ZnCl_2_ helps increase the content of carbohydrates and decrease the contents of ketones, phenols, alcohols, esters, and N-compounds; when applying HZSM-5 as the catalyst, hydrocarbons and alcohols reach their highest contents while acids have the lowest content; MCM-41 promotes the generation of aldehydes and furans while inhibiting that of carbohydrates. 

Due to the wide variety, complex structure, and diverse pyrolysis products of biomass, there remain many challenges in the industrial application of bio-oil. AW and RH, as typical forestry and agricultural residues, can provide some references for the thermal conversion and utilization of agricultural and forestry wastes. To achieve a high-value utilization of biomass, the pyrolysis mechanism of each component of the biomass, the distribution of pyrolysis products of active and passive biomass and the co-pyrolysis of biomass and waste plastics could be studied in the future. In addition, the preparation of various modified catalysts as well as their influence on the pyrolysis products warrants further investigation to understand more thoroughly the pyrolysis of biomass and promote its industrial application.

## Figures and Tables

**Table 1:** Sample abbreviations and Zn^2+^ content.

Sample	Abbreviation	Zn^2+^ Content (%)
AW:HZSM-5 = 3:1	AWH31	-
AW:HZSM-5 = 1:1	AWH11	-
AW:HZSM-5 = 1:3	AWH13	-
AW:MCM-41 = 3:1	AWM31	-
AW:MCM-41 = 1:1	AWM11	-
AW:MCM-41 = 1:3	AWM13	-
AW + ZnCl_2_ 30 mg	AWZn30	0.48
AW + ZnCl_2_ 75 mg	AWZn75	0.77
AW + ZnCl_2_ 150 mg	AWZn150	1.09
AW + ZnCl_2_ 300 mg	AWZn300	1.29
RH:HZSM-5 = 3:1	RHH31	-
RH:HZSM-5 = 1:1	RHH11	-
RH:HZSM-5 = 1:3	RHH13	-
RH:MCM-41 = 3:1	RHM31	-
RH:MCM-41 = 1:1	RHM11	-
RH:MCM-41 = 1:3	RHM13	-
RH + ZnCl_2_ 30 mg	RHZn30	0.44
RH + ZnCl_2_ 75 mg	RHZn75	0.74
RH + ZnCl_2_ 150 mg	RHZn150	1.02
RH + ZnCl_2_ 300 mg	RHZn300	1.22

**Table 2:** Chemical analyzes of lignocellulosic biomass.

Sample	Proximate Analysis (wt %)				Ultimate Analysis (wt %, Dry Basis)					Component Analysis (wt %, Dry Basis)		
	Moisture	Volatile	Fixed Carbon	Ash	C	H	O	N	S	Cellulose	Hemicellulose	Lignin
AW	11.46	76.23	11.53	0.78	46.77	6.75	45.41	0.35	0.06	47.96	14.49	25.56
RH	10.02	68.28	13.68	8.02	41.03	5.42	44.54	0.86	0.13	35.87	33.16	13.84

## Supplementary Materials

The following supporting information can be downloaded at: https://www.mdpi.com/article/10.3390/polym15143104/s1, Table S1: The product distributions from the non-catalytic pyrolysis of AW at different temperatures; Table S2: The product distributions from the non-catalytic pyrolysis of RH at different temperatures.
