# Supplementary material for: Influences of the Reaction Temperature and Catalysts on the Pyrolysis Product Distribution of Lignocellulosic Biomass (Aspen Wood and Rice Husk)
Source: Polymers (Basel). 2023 Jul 21;15(14):3104. doi: 10.3390/polym15143104 (PMC10383021; doi:10.3390/polym15143104)
Supplement: Supplementary file 1 [file polymers-15-03104-s001.zip › polymers-2452897-supplementary.pdf]

Table S1 The product distributions from the non-catalytic pyrolysis of AW at different temperatures

Category	Compounds	285℃		345℃		445℃		500℃		600℃		700℃	
		Peak area	Peak area /%	Peak area	Peak area /%	Peak area	Peak area /%	Peak area	Peak area /%	Peak area	Peak area /%	Peak area	Peak area /%
Aldehydes	Acetaldehyde	-	-	-	-	-	-	-	-	3.32×10^{6}	4.15	4.91×10^{6}	9.42
	Methylglyoxal	-	-	-	-	3.84×10^{6}	4.23	4.41×10^{6}	4.54	4.80×10^{6}	6.00	4.33×10^{6}	8.31
	Succindialdehyde	-	-	-	-	1.12×10^{6}	1.23	1.79×10^{6}	1.84	1.14×10^{6}	1.43	5.32×10^{5}	1.02
	Furfural	-	-	-	-	1.15×10^{6}	1.27	9.88×10^{5}	1.02	9.80×10^{5}	1.22	4.38×10^{5}	0.84
	Nonanal	3.68×10^{4}	1.33	1.37×10^{5}	2.08	-	-	-	-	-	-	-	-
	2,3-Dihydroxybenzaldehyde	-	-	-	-	6.42×10^{5}	0.71	5.24×10^{5}	0.54	7.44×10^{5}	0.93	-	-
	Vanillin	-	-	4.98×10^{4}	0.75	6.91×10^{5}	0.76	5.78×10^{5}	0.59	6.39×10^{5}	0.80	-	-
	Benzaldehyde,4-hydroxy-3,5-dimethoxy-	-	-	-	-	7.94×10^{5}	0.88	7.77×10^{5}	0.79	6.69×10^{5}	0.84	-	-
Acids	Acetic acid	1.84×10^{5}	6.65	1.08×10^{6}	16.30	1.36×10^{7}	15.02	1.40×10^{7}	14.43	7.10×10^{6}	8.87	8.67×10^{6}	16.66
	4-Hydroxybenzoic acid	-	-	-	-	2.77×10^{6}	3.06	1.74×10^{6}	1.79	1.50×10^{6}	1.88	-	-
	Palmitic acid	2.31×10^{5}	8.35	2.32×10^{5}	3.51	1.08×10^{6}	1.19	7.95×10^{5}	0.82	6.90×10^{5}	0.86	-	-
	Oleic Acid	3.26×10^{5}	11.76	3.59×10^{5}	5.44	1.60×10^{6}	1.76	5.33×10^{5}	0.55	-	-	-	-
	Stearic acid	8.25×10^{4}	2.98	1.22×10^{5}	1.84	-	-	-	-	-	-	-	-
Alcohols	2-Amino-1,3-propanediol	-	-	-	-	-	-	-	-	1.51×10^{6}	1.89	9.22×10^{5}	1.77
	4-Penten-2-ol	-	-	-	-	-	-	-	-	-	-	1.57×10^{6}	3.01
	2-Furanmethanol	-	-	1.60×10^{5}	2.42	6.19×10^{5}	0.68	-	-	-	-	-	-
	2-Cyclohexen-1-ol	-	-	-	-	7.86×10^{5}	0.87	6.62×10^{5}	0.68	-	-	-	-
	Cyclopropyl carbinol	-	-	1.00×10^{5}	1.51	2.31×10^{6}	2.55	1.77×10^{6}	1.82	1.03×10^{6}	1.29	-	-
	trans-Sinapyl alcohol	-	-	2.64×10^{5}	3.99	8.00×10^{6}	8.82	1.09×10^{7}	11.18	1.34×10^{6}	1.67	-	-
Ketones	2,3-Butanedione	-	-	-	-	-	-	-	-	5.98×10^{6}	7.48	-	-
	Hydroxyacetone	-	-	2.54×10^{5}	3.85	5.27×10^{6}	5.82	7.54×10^{6}	7.75	7.18×10^{6}	8.98	3.97×10^{6}	7.62
	2(5H)-Furanone	-	-	-	-	7.39×10^{5}	0.82	1.01×10^{6}	1.04	9.59×10^{5}	1.20	-	-
	5-Methylfuran-2(3H)-one	-	-	1.59×10^{5}	2.41	3.08×10^{6}	3.39	3.09×10^{6}	3.18	3.02×10^{6}	3.78	1.51×10^{6}	2.90
	1,2-Cyclopentanedione,3-methyl-	-	-	-	-	8.83×10^{5}	0.97	9.64×10^{5}	0.99	9.68×10^{5}	1.21	4.42×10^{5}	0.85
	1,4-Dioxaspiro[2.4]heptan-5-one	-	-	-	-	6.49×10^{5}	0.72	6.80×10^{5}	0.70	6.83×10^{5}	0.85	-	-
	Apocynin	-	-	-	-	5.89×10^{5}	0.65	8.90×10^{5}	0.91	-	-	-	-
Phenols	Phenol	-	-	1.14×10^{5}	1.72	2.31×10^{6}	2.54	2.42×10^{6}	2.49	2.73×10^{6}	3.41	2.00×10^{6}	3.83
	p-Cresol	-	-	-	-	-	-	-	-	6.42×10^{5}	0.80	1.12×10^{6}	2.14

	Guaiacol	-	-	-	-	8.48×10^5	0.93	1.31×10^6	1.35	8.69×10^5	1.09	-	-
	2-Methoxy-4-methylphenol	-	-	-	-	-	-	6.48×10^5	0.67	-	-	-	-
	Catechol	-	-	-	-	8.78×10^5	0.97	9.10×10^5	0.94	1.27×10^6	1.59	1.19×10^6	2.28
	3-Methylcatechol	-	-	-	-	-	-	-	-	7.88×10^5	0.99	6.22×10^5	1.19
	1,2-Benzenediol, 3-methoxy-	-	-	-	-	6.64×10^5	0.73	8.77×10^5	0.90	8.32×10^5	1.04	-	-
	1,2-Benzenediol, 4-methyl-	-	-	-	-	-	-	7.10×10^5	0.73	1.16×10^6	1.45	1.14×10^6	2.19
	4-Ethylcatechol	-	-	-	-	-	-	-	-	-	-	1.04×10^6	2.00
	2-Methoxy-4-vinylphenol	-	-	6.54×10^4	0.99	1.61×10^6	1.78	2.00×10^6	2.05	1.42×10^6	1.78	-	-
	Syringol	-	-	-	-	2.15×10^6	2.38	2.44×10^6	2.51	1.79×10^6	2.25	-	-
	cis-Isoeugenol	-	-	7.63×10^4	1.15	1.40×10^6	1.54	1.28×10^6	1.31	9.67×10^5	1.21	-	-
	Vinylsyringol	-	-	9.15×10^4	1.39	3.12×10^6	3.45	3.15×10^6	3.24	1.73×10^6	2.16	-	-
	Phenol,2,6-dimethoxy-4-(2-propenyl)-	-	-	-	-	6.15×10^5	0.68	6.34×10^5	0.65	-	-	-	-
	4-((1E)-3-Hydroxy-1-propenyl)-2-methoxyphenol	-	-	8.97×10^5	13.57	8.35×10^6	9.21	1.03×10^7	10.58	3.08×10^6	3.85	-	-
	trans-Propenylsyringol	-	-	4.64×10^4	0.70	1.55×10^6	1.71	1.62×10^6	1.66	6.86×10^5	0.86	-	-
Carbohydrates	3,6-Dianhydro-alpha-glucopyranose	-	-	-	-	-	-	5.46×10^5	0.56	7.39×10^5	0.92	-	-
	Levoglucosan	2.12×10^4	0.77	1.10×10^5	1.66	2.15×10^6	2.36	2.51×10^6	2.58	3.25×10^6	4.07	1.53×10^6	2.93
Hydrocarbons	1,3-Cyclopentadiene	-	-	-	-	-	-	-	-	7.16×10^5	0.90	1.45×10^6	2.79
	Methylcyclopentadiene	-	-	-	-	-	-	-	-	-	-	6.10×10^5	1.17
	Cyclohexadiene	-	-	-	-	-	-	-	-	-	-	1.18×10^6	2.27
	Isopropylcyclobutane	-	-	-	-	-	-	-	-	-	-	4.70×10^5	0.90
	Toluene	-	-	-	-	-	-	-	-	-	-	2.77×10^6	5.32
	Ethylbenzene	-	-	-	-	-	-	-	-	-	-	7.35×10^5	1.41
	Styrene	-	-	-	-	-	-	-	-	-	-	6.62×10^5	1.27
	Indene	-	-	-	-	-	-	-	-	-	-	7.18×10^5	1.38
	Naphthalene	-	-	-	-	-	-	-	-	-	-	3.44×10^5	0.66
	Tetradecane	3.43×10^4	1.24	-	-	-	-	-	-	-	-	-	-
Esters	Methyl acrylate	-	-	9.24×10^4	1.40	1.39×10^6	1.54	1.02×10^6	1.05	-	-	-	-
	2-Hydroxyethyl acetate	-	-	-	-	2.82×10^6	3.11	3.12×10^6	3.21	3.14×10^6	3.92	5.31×10^5	1.02
	Methyl pyruvate	-	-	-	-	1.73×10^6	1.91	1.96×10^6	2.02	2.20×10^6	2.75	6.48×10^5	1.24
	2,2,4-Trimethyl-1,3-pentanediol diisobutyrate	5.30×10^4	1.91	8.25×10^4	1.25	-	-	-	-	-	-	-	-

	Dibutyl phthalate	5.02×10^{4}	1.81	4.66×10^{4}	0.71	-	-	-	-	-	-	-	-
	Bis(2-ethylhexyl) phthalate	3.75×10^{5}	13.59	4.37×10^{5}	6.62	8.43×10^{5}	0.93	-	-	-	-	-	-
Furans	7-Methylbenzofuran	-	-	-	-	-	-	-	-	-	-	1.02×10^{6}	1.95
Ethers	Ethyl vinyl ether	-	-	2.50×10^{5}	3.79	-	-	-	-	-	-	-	-
N-compounds	Oxazolidine,2,2-diethyl-3-methyl-	-	-	8.68×10^{4}	1.31	1.25×10^{6}	1.37	9.83×10^{5}	1.01	7.82×10^{5}	0.98	3.83×10^{5}	0.73
	Erucamide	3.01×10^{5}	10.87	3.12×10^{5}	4.73	-	-	-	-	-	-	-	-

Table S2 The product distributions from the non-catalytic pyrolysis of RH at different temperatures

Category	Compounds	285℃		345℃		445℃		500℃		600℃		700℃	
		Peak area	Peak area /%	Peak area	Peak area /%	Peak area	Peak area /%	Peak area	Peak area /%	Peak area	Peak area /%	Peak area	Peak area /%
Aldehydes	Methylglyoxal	-	-	9.53×10^{5}	2.71	4.18×10^{6}	4.40	4.77×10^{6}	4.91	4.99×10^{6}	5.88	-	-
	Hexanal	2.56×10^{5}	1.18	2.03×10^{5}	0.58	-	-	-	-	-	-	-	-
	Crotonaldehyde	-	-	-	-	-	-	-	-	-	-	5.32×10^{5}	0.76
	Succindialdehyde	-	-	-	-	8.22×10^{5}	0.87	1.15×10^{6}	1.18	8.59×10^{5}	1.01	7.63×10^{5}	1.09
	Furfural	-	-	1.44×10^{5}	0.41	7.62×10^{5}	0.80	9.10×10^{5}	0.94	6.97×10^{5}	0.82	4.18×10^{5}	0.60
	Nonanal	1.75×10^{5}	0.81	1.03×10^{5}	0.29	-	-	-	-	-	-	-	-
	5-Hydroxymethylfurfural	-	-	2.36×10^{5}	0.96	5.38×10^{5}	0.57	4.54×10^{5}	0.47	-	-	-	-
Acids	Acetic acid	1.86×10^{5}	0.86	1.60×10^{6}	4.55	7.31×10^{6}	7.69	7.30×10^{6}	7.52	5.67×10^{6}	6.68	6.74×10^{6}	9.61
	L-Lactic acid	-	-	-	-	-	-	-	-	1.15×10^{6}	1.36	1.89×10^{6}	2.69
	Hexanoic acid	2.22×10^{5}	1.03	2.55×10^{5}	0.73	6.06×10^{5}	0.64	-	-	-	-	-	-
	Nonanoic acid	8.21×10^{4}	0.38	1.04×10^{5}	0.30	4.54×10^{5}	0.48	-	-	8.02×10^{5}	0.95	-	-
	6-Heptenoic acid	-	-	-	-	-	-	-	-	-	-	1.01×10^{6}	1.44
	Azelaic acid	-	-	-	-	5.35×10^{5}	0.56	4.55×10^{5}	0.47	5.10×10^{5}	0.60	-	-
	Palmitic acid	4.74×10^{6}	22.03	5.63×10^{6}	16.01	1.08×10^{7}	11.33	9.88×10^{6}	10.17	9.67×10^{6}	11.40	2.63×10^{6}	3.75
	Oleic Acid	7.40×10^{6}	34.34	1.25×10^{7}	35.64	2.66×10^{7}	27.99	1.52×10^{7}	15.66	1.33×10^{7}	15.70	2.04×10^{6}	2.92
	Stearic acid	2.48×10^{5}	1.15	2.69×10^{5}	0.77	4.48×10^{5}	0.47	2.00×10^{6}	2.06	1.92×10^{6}	2.26	-	-
	9,12-Octadecadienoic acid (Z,Z)-	-	-	-	-	-	-	1.04×10^{6}	1.07	1.56×10^{6}	1.84	-	-
Alcohols	1-Pentanol, 2-methyl-	-	-	-	-	-	-	-	-	-	-	7.10×10^{6}	10.12
	Glycidol	-	-	-	-	-	-	-	-	3.29×10^{6}	3.88	-	-
	Oleic Acid	-	-	-	-	-	-	-	-	1.38×10^{6}	1.63	2.05×10^{6}	2.92
	2-Furanmethanol	-	-	2.68×10^{5}	0.76	8.43×10^{5}	0.89	8.50×10^{5}	0.88	-	-	-	-

	2-Cyclohexen-1-ol	-	-	-	-	7.00×10^{5}	0.74	6.89×10^{5}	0.71	-	-	-	-
	Glycerin	1.85×10^{5}	0.86	6.82×10^{5}	1.94	4.46×10^{5}	0.47	-	-	-	-	-	-
	Cyclopropyl carbinol	-	-	-	-	2.00×10^{6}	2.11	1.43×10^{6}	1.47	7.42×10^{5}	0.87	-	-
	1,3-Propanediol, 2-ethyl-2-(hydroxymethyl)-	1.97×10^{5}	0.91	1.03×10^{6}	2.94	2.44×10^{6}	2.56	2.03×10^{6}	2.09	-	-	-	-
Ketones	Hydroxyacetone	-	-	4.70×10^{5}	1.34	4.00×10^{6}	4.21	6.79×10^{6}	6.98	3.90×10^{6}	4.60	4.40×10^{6}	6.28
	2-Cyclopentenone	-	-	7.62×10^{4}	0.22	9.85×10^{5}	1.04	1.38×10^{6}	1.42	6.07×10^{5}	0.72	4.28×10^{5}	0.61
	5-Methylfuran-2(3H)-one	-	-	1.55×10^{5}	0.44	2.02×10^{6}	2.12	2.46×10^{6}	2.53	1.54×10^{6}	1.81	1.11×10^{6}	1.58
	1,2-Cyclopentanedione, 3-methyl-	-	-	-	-	5.89×10^{5}	0.62	8.85×10^{5}	0.91	7.43×10^{5}	0.88	5.52×10^{5}	0.79
Phenols	Phenol	-	-	-	-	-	-	6.98×10^{5}	0.72	7.98×10^{5}	0.94	1.11×10^{6}	1.58
	p-Cresol	-	-	-	-	-	-	-	-	8.27×10^{5}	0.97	1.16×10^{6}	1.65
	Guaiacol	-	-	-	-	6.05×10^{5}	0.64	1.63×10^{6}	1.67	-	-	-	-
	Catechol	-	-	-	-	-	-	8.04×10^{5}	0.83	7.59×10^{5}	0.89	7.28×10^{5}	1.04
	4-Vinylphenol	2.29×10^{5}	1.06	1.02×10^{6}	2.91	2.90×10^{6}	3.05	3.51×10^{6}	3.61	2.03×10^{6}	2.40	1.88×10^{6}	2.68
	1,2-Benzenediol, 3-methoxy-	-	-	-	-	-	-	4.84×10^{5}	0.50	-	-	4.90×10^{5}	0.70
	Hydroquinone	-	-	-	-	-	-	4.62×10^{5}	0.48	5.00×10^{5}	0.59	-	-
	2-Methoxy-4-vinylphenol	7.08×10^{4}	0.33	4.75×10^{5}	1.35	3.28×10^{6}	3.46	2.51×10^{6}	2.58	1.27×10^{6}	1.50	-	-
	Syringol	-	-	-	-	5.10×10^{5}	0.54	6.21×10^{5}	0.64	-	-	-	-
	cis-Isoeugenol	-	-	8.06×10^{4}	0.23	5.00×10^{5}	0.53	7.34×10^{5}	0.76	-	-	-	-
	Vinylsyringol	-	-	9.78×10^{4}	0.28	6.30×10^{5}	0.66	7.66×10^{5}	0.79	6.48×10^{5}	0.76	-	-
	4-((1E)-3-Hydroxy-1-propenyl)-2-methoxyphenol	-	-	8.55×10^{5}	2.43	1.82×10^{6}	1.92	2.03×10^{6}	2.09	8.43×10^{5}	0.99	-	-
Carbohydrates	Levoglucosan	-	-	1.22×10^{5}	0.35	2.60×10^{6}	2.74	5.86×10^{6}	6.04	3.46×10^{6}	4.08	3.05×10^{6}	4.35
Hydrocarbons	1-Butene	-	-	-	-	-	-	-	-	-	-	7.23×10^{6}	10.31
	1-Butyne, 3-methyl-	-	-	-	-	-	-	-	-	-	-	9.85×10^{5}	1.40
	1-Penten-3-yne	-	-	-	-	-	-	-	-	6.37×10^{5}	0.75	2.21×10^{6}	3.15
	Cyclohexadiene	-	-	-	-	-	-	-	-	-	-	2.27×10^{6}	3.23
	Cyclohexene	-	-	-	-	-	-	-	-	-	-	1.08×10^{6}	1.54
	1-Heptene	-	-	-	-	-	-	-	-	1.22×10^{6}	1.43	1.77×10^{6}	2.53
	Toluene	-	-	-	-	-	-	-	-	-	-	2.18×10^{6}	3.10
	Ethylbenzene	-	-	-	-	-	-	-	-	-	-	5.57×10^{5}	0.79
	1,8-Nonadiene	-	-	-	-	-	-	-	-	5.99×10^{5}	0.71	4.57×10^{5}	0.65
	Styrene	-	-	-	-	-	-	-	-	-	-	7.79×10^{5}	1.11

	1-Decene	-	-	-	-	-	-	-	-	5.04×10^{5}	0.59	6.22×10^{5}	0.89
	1-Undecene	-	-	-	-	-	-	-	-	6.39×10^{5}	0.75	5.25×10^{5}	0.75
	9-Octadecene, (E)-	-	-	-	-	-	-	-	-	5.23×10^{5}	0.62	4.68×10^{5}	0.67
Esters	Methyl acrylate	-	-	2.20×10^{5}	0.63	1.15×10^{6}	1.21	5.36×10^{5}	0.55	-	-	-	-
	2-Hydroxyethyl acetate	-	-	-	-	1.33×10^{6}	1.40	1.80×10^{6}	1.85	-	-	-	-
	Methyl pyruvate	-	-	-	-	1.21×10^{6}	1.28	1.78×10^{6}	1.84	1.73×10^{6}	2.03	1.14×10^{6}	1.62
	9-Octadecenoic acid, 1,2,3-propanetriyl ester, (E,E,E)-	-	-	-	-	-	-	5.77×10^{5}	0.59	1.53×10^{6}	1.80	-	-
	Bis(2-ethylhexyl) phthalate	4.95×10^{5}	2.30	2.64×10^{5}	0.75	4.64×10^{5}	0.49	6.37×10^{5}	0.66	-	-	-	-
Furan	Furan, 2,5-dihydro-	-	-	-	-	-	-	-	-	-	-	7.68×10^{5}	1.10
N-compounds	Propanenitrile, 2,2-dimethyl-	-	-	-	-	-	-	-	-	8.52×10^{5}	1.00	5.83×10^{5}	0.83
	Aziridine, 2-methyl-3-(1-methylethyl)-, trans-	-	-	-	-	-	-	1.02×10^{6}	1.05	9.02×10^{5}	1.06	7.33×10^{5}	1.04
	Oxazolidine, 2,2-diethyl-3-methyl-	-	-	-	-	6.00×10^{5}	0.63	6.11×10^{5}	0.63	-	-	-	-
	Erucamide	5.69×10^{6}	26.42	5.16×10^{6}	14.67	5.37×10^{6}	5.66	4.62×10^{6}	4.75	3.81×10^{6}	4.49	1.99×10^{6}	2.84
